# Explorations of the convergence of narrative therapy with psychoanalysis

**DOI:** 10.1192/j.eurpsy.2025.1937

**Published:** 2025-08-26

**Authors:** L. Mehl-Madrona, B. Mainguy

**Affiliations:** 1 Native Studies, University of Maine, Orono; 2 Psychiatry Residency, Northern Light Acadia; 3 Wabanaki Health and Wellness, Bangor, United States

## Abstract

**Introduction:**

Two seemingly different paradigms for psychotherapy appear to have converged in narrative psychotherapy and psychoanalysis.

**Objectives:**

We wanted to explore the philosophical perspectives of some of their practitioners to understand this seemingly emergent paradigm.

**Methods:**

We interviewed narrative psychotherapists and practitioners identifying as narrative psychoanalysts about their philosophical foundations in order to explore this apparent convergence. We used constructivist grounded theory to produce common themes. We also put the interviews through an AI program -- Perplexity to explore how well it correlated with our non-AI analysis.

**Results:**

Both groups of practitioners believed that that we construct stories to give us identity and meaning in our lives. The practitioners who came from a psychoanalytic perspective described their coming to understand that psychoanalysis is embedded in stories that ciients and therapists tell. Patients and analysts bring, create, and modify stories in analytic work. Both types of practitioners described the importance of cultivating an active listening attitude on the part of the clinician. Jacques Lacan was often quoted as saying that the greatest gift we can give our clients is to listen without judgment or interpretation. Narrative therapists were more inclined to mention Hermans’ dialogical self-theory while psychoanalytically trained therapists spoke of object relations theory with both supporting a philosophy of making meaning together. Both groups of practioners mentioned the importance of meaning being constructed within a particular context. Both groups mentioned the importance of reflective practices, Buddhism, and Indigenous philosophies to support the introspective elements of this technique along with therapy being grounded in our relationships with others. Both groups emphasized the importance of showing how creating new life narratives over time transforms our sense of self, relationship, and meaning.

**Conclusions:**

Qualitative analysis of these two groups of practitioners’ philosophies of psychotherapy supports the idea of a convergence between narrative psychotherapy and psychoanalysis which may promise to enrich both groups.

**Disclosure of Interest:**

None Declared

